# Liver-first approach to the treatment of patients with synchronous colorectal liver metastases: a systematic review and meta-analysis

**DOI:** 10.31744/einstein_journal/2024RW0596

**Published:** 2024-11-12

**Authors:** Bruno Mirandola Bulisani, Milena Arruda de Oliveira Leite, Jaques Waisberg

**Affiliations:** 1 Centro Universitário FMABC Santo André SP Brazil Centro Universitário FMABC, Santo André, SP, Brazil.

**Keywords:** Colorectal neoplasms, Neoplasm metastasis, Liver neoplasms, Liver surgery, Hepatectomy

## Abstract

**Objective:**

The optimal approach to the treatment of colorectal carcinoma and synchronous liver metastases remains controversial. The objective of this review was to analyze the outcomes of adopting the liver-first approach for the treatment of patients with colorectal cancer with synchronous hepatic metastases who initially underwent systemic chemotherapy and/or resection of the metastatic lesions and primary colorectal carcinoma.

**Methods:**

This review was conducted in accordance with the Preferred Reporting Items for Systematic Reviews and Meta-Analyses guidelines. The MEDLINE, EMBASE, LILACS, and Cochrane Central Register of Controlled Trials databases were searched for the identification and retrieval of eligible studies. Studies that included details of using the liver-first approach for the treatment of synchronous liver metastases of colorectal cancer and its outcomes, including the patients’ survival data, were included. Proportional meta-analysis was performed using the random-effects restricted maximum likelihood method to summarize the three- and five-year overall survival and recurrence rates of the patients.

**Results:**

Eight hundred and fifty-five articles describing the results of studies on the liver-first approach were identified. Three independent reviewers screened the titles and abstracts of the articles and excluded 750 articles. Thereafter, 29 retrospective and comparative studies that met the inclusion criteria were included. No randomized controlled trials were identified in the database search.

**Conclusion:**

Neoadjuvant treatment with systemic chemotherapy for hepatic metastasis can prepare a patient for resection of liver metastases, offering the opportunity for potentially curative treatment of synchronous hepatic metastases initially considered unresectable. The decision regarding the resection of primary colorectal carcinoma and liver metastases should be based on individualized patient response. Prospero database registration ID: CRD42022337047 (www.crd.york.ac.uk/prospero).

## INTRODUCTION

Colorectal cancer (CRC) is the third most common cancer worldwide^(1)^ and has the fourth highest mortality rate of all cancers.^(2)^ Colorectal liver metastases (CLM), which occur in up to 25% of CRC cases, are considered synchronous when diagnosed before or at the same time as the primary cancer, or up to 6 months after the detection of the primary cancer.^(3-5)^ This presentation of the disease is strongly associated with a low survival rate relative to that of patients with metastatic liver disease.^(3,4)^Given that most patients with synchronous CLM have unresectable disease, they tend to have a more aggressive cancer biology and lower probability of long-term survival.^(6,7)^

Apart from systemic chemotherapy, the most appropriate approach to the treatment of synchronous CRC and CLM remains controversial.^(7)^ Complete resection of neoplasms offers a five-year survival rate of up to 58%;^(7,8)^ however, only 25% of patients with synchronous CLM are candidates for radical oncological resection.^(7-10)^ The conventional approach to the treatment of patients with resected synchronous CLM involves two stages: resection of the CRC, followed by chemotherapy and resection of the CLM.^(11)^ However, the main disadvantage of this approach is that the attempt to control the CRC, especially in the context of postponing systemic treatment to prevent morbidity associated with colorectal resection and/or adjuvant chemotherapy,^(12,13)^ provides an opportunity for progression of the CLM to the point of unresectability, thus preventing the initiation of systemic chemotherapy focused on CLM and its resection.

Simultaneous resection of CRC and CLM is being increasingly performed for selected patients with acceptable preoperative morbimortality and survival outcomes.^(14,15)^However, this method is associated with an increase in postoperative complications when a broader hepatic CLM resection is performed.^(16,17)^ Although this procedure may be advantageous in terms of shorter duration of surgery and lower hospital admission costs, it is not feasible for patients who have a high CLM burden and require large liver resection, or for elderly patients with locally advanced rectal cancer.^(18-20)^

The best sequence for hepatic CLM resection and systemic chemotherapy in patients with synchronous CLM remains controversial and has not been defined to date.^(3,4,7,10,12,21-23)^ The hypothesis that hepatic metastasis is the most important risk factor for death in patients with synchronous CLM was proposed by Mentha et al.,^(24)^ who described the liver-first approach as a regimen that includes systemic chemotherapy focused on liver metastases for the achievement of tumor downstaging, followed by resection of the CLM and subsequent resection of the primary CRC. Initially, this approach was indicated for patients with synchronous CLM of rectal cancer who required adjuvant chemoradiotherapy. The key reason for choosing the liver-first approach for the treatment of synchronous liver metastases of CRC is that it allows for control of the CLM, optimizing the chance of a potentially curative liver resection, which can improve the patient’s chances of survival.^(25,26)^

Proper patient selection is essential for the chronological and successful treatment of synchronous CLM to ensure the achievement of optimal perioperative and long-term oncological outcomes. The factors essential for determining whether a patient is a candidate for liver resection include factors related to the disease, the patient (oncological criteria), and the anatomy of the lesions (technical/surgical criteria).^(27-29)^

Several studies have demonstrated that the liver-first approach is a viable option for the treatment of patients with synchronous liver metastases of CRC (Table 1).^(29-57)^ Adopting the liver-first approach for tumor downstaging includes the administration of systemic chemotherapy and/or chemoradiotherapy, followed by resection of the CLM before resection of the CRC.^(30,33,36,38)^ The primary objective of this approach is to control the synchronous hepatic metastases of the CRC, thus improving the chances of a potentially curative liver resection and achieving satisfactory long-term survival outcomes.^(32,34,35)^

**Table 1:** Mean epidemiological characteristics of patients with synchronous liver metastases of colorectal cancer treated using the liver first approach

	Age	Sex (%)	Primary site (%)	Patients with extrahepatic metastasis, n (%)	Average diameter of the metastasis	CEA level (ng/ml)
Esposito et al.^(29)^ (n=66)	60.3 (49–71)	F = 40.9 M = 59.1	R = 29 (44) C = 37 (56)	-	4.1 cm	812.36
Mentha et al.^(30)^ (n=35)	52 (32–69)	F = 46.7 M = 53.3	R = 13 (44.4) C = 17 (56.6)	3 (8.5)	6 cm	48
Brouquet et al.^(31)^ (n=27)	48 (25–78)	F = 63 M = 37	R = 19 (70.3) C = 8 (29.7)	NA	4 cm	34
van der Pool et al.^(32)^ (n=20)	61 (43–82)	-	R = 20 (100) C = 0 (0)	NA	NA	NA
de Jong et al.^(33)^ (n=22)	65 (41–86)	F = 27.2 M = 72.7	R = 19 (86.4) C = 3 (13.6)	NA	1.7 cm	15.8
Ayez et al.^(34)^ (n=42)	61 (42–78)	F = 21.5 M = 78.5	R = 42 (100) C = 0 (0)	4 (9.5)	2.7 cm	41
Mayo et al.^(35)^ (n=28)	58 (46–70)	F = 39.2 M = 60.7	R = 15 (53.6) C = 13 (46.4)	1 (3.6)	3 cm	NA
de Rosa et al.^(36)^ (n=37)	65 (25–73)	F = 29.7 M = 70.2	R = 25 (67.5) C = 12 (32.5)	-	NA	NA
Buchs et al.^(37)^ (n=34)	57 (38–78)	F = 42.4 M = 57.6	R = 34 (100) C = 0 (0)	-	3 cm	21.4
Sabbagh et al.^(38)^ (n=10)	59	F = 20 M = 80	R = 10 (100) C = 0 (0)	-	NA	28.9
Tanaka et al.^(39)^ (n=10)	63.5 (39–74)	F = 50 M = 50	R = 2 (20) C = 8 (80)	1 (10)	5.3 cm	29.9
Okuno et al.^(40)^ (n=12)	58 (36–69)	-	R = 7 (58.3) C = 5 (41.7)	6 (50)	5.7 cm	105.5
Wang et al.^(41)^ (n=18)	54 (21–74)	F = 44.4 M = 55.5	R = 16 (88.8) C = 2 (11.2)	NA	4 cm	26.3
Welsh et al.^(42)^ (n=98)	61 (50–70,1)	F = 38.7 M = 61.2	R = 44 (44.9) C = 54 (55.1)	NA	3 cm	NA
Valdimarsson et al.^(43)^ (n=246)	62 (54–69)	F = 34.6 M = 65.4	R = 166 (67.4) C = 80 (32.6)	NA	NA	NA
Nierop et al.^(44)^ (n=129)	62 (56–68)	F = 28.7 M = 71.3	R = 129 (100) C = 0 (0)	19 (14.7)	3.85 cm	53.15
de Jong et al.^(45)^ (n=92)	65 (30–86)	F = 23.9 M = 76.1	R = 68 (73.9) C = 24 (26.1)	6 (6.5)	2.5 cm	NA
Giuliante et al.^(46)^ (n=552)	N.A	F = 37.1 M = 62.9	R = 317 (58) C = 230 (42)	35 (6.3)	NA	NA
Fonollosa et al.^(47)^ (n=88)	61 (32-80)	F = 38.6 M = 61.4	R = 31 (35.2) C = 57 (64.7)	14 (15.9)	4.27cm	163.8 (1–1621)
Carbone et al.^(48)^ (n=26)	57 (54-65)	F = 26.9 M = 73.1	R = 13 (50) C = 13 (50)	5 (19.2)	NA	NA
Frühling et al.^(49)^ (n=163)	65.1	F = 39 M = 61	R = 108 (66.3) C = 55 (33.7)	NA	30mm	NA
Raoux et al.^(50)^ (n=26)	59 (49 – 69)	F = 38 M = 62	R = 5 (19.2) C = 21(80.8)	NA	NA	NA
Reding et al.^(51)^ (n=7)	54.5 (48 – 66)	F = 29 M = 71	R = 5 (71) C = 2 (29)	4 (58)	NA	NA
Harufumi et al.^(52)^ (n=141)	54 (43–63)	F = 43.2 M = 56.7	R = 28 C = 113	29 (20.6)	2.3 cm	83
Giammauro et al.^(53)^ (n=62)	66.6 (49 – 71)	F = 35.5 M = 64.5	R = 47 (76) C = 15 (24)	-	5.42 cm	25 (2 - 1282)
Vallance et al.^(54)^ (n=270)	NA	F = 35.9 M = 64.1	R = 152 C = 118	-	NA	NA
Ramia et al.^(55)^ (n=149)	61 (52 – 68)	F = 35.5 M = 64.5	R = 72 C = 77	NA	3 cm	NA
Labori et al.^(56)^ (n=45)	62 (33 – 73)	F = 53.3 M = 46.7	R = 45 C = 0	1 (2.2)	2.4 cm	NA
Pasquier et al.^(57)^ (n=44)	63 (23 – 78)	F = 36.4 M = 63.6	R = 19 C = 25	3 (7)	5 cm	24.5

n: number of patients; M: male; F: female; R: rectum; C: colon; CEA: carcinoembryonic antigen; NA: not available.

Metastatic disease seems to be the most important factor that affects patient survival; therefore, the treatment of CLM in patients with CRC should be a priority.^(30,33,34,36)^Patients with unresectable CLM who respond to chemotherapy should be re-evaluated periodically for resectability. In addition, relatively minor changes in CLM size, particularly at critical sites, may have significant implications for the technical viability of the section.^(37-39)^

The liver-first approach may be an option for patients with early stage CRC and disseminated metastatic liver disease or patients with locally advanced CRC with limited or extensive liver disease.^(37,40,42)^Appropriate patient selection is crucial for the achievement of the best possible survival outcomes using this method. The decision to perform surgery is based on the patient’s response to neoadjuvant therapy and the burden of liver disease.^(36)^ Thus, there is a clear need for an effective neoadjuvant treatment that provides high tumor response rates, leading to improved resectability and offering an opportunity for potentially curative resection of unresectable CLM.^(37,41)^

The aim of this systematic review and meta-analysis was to evaluate the outcomes of the liver-first approach in patients with synchronous CLM that was initially considered unresectable.

## METHODS

### Literature search strategy

**Figure 1:**
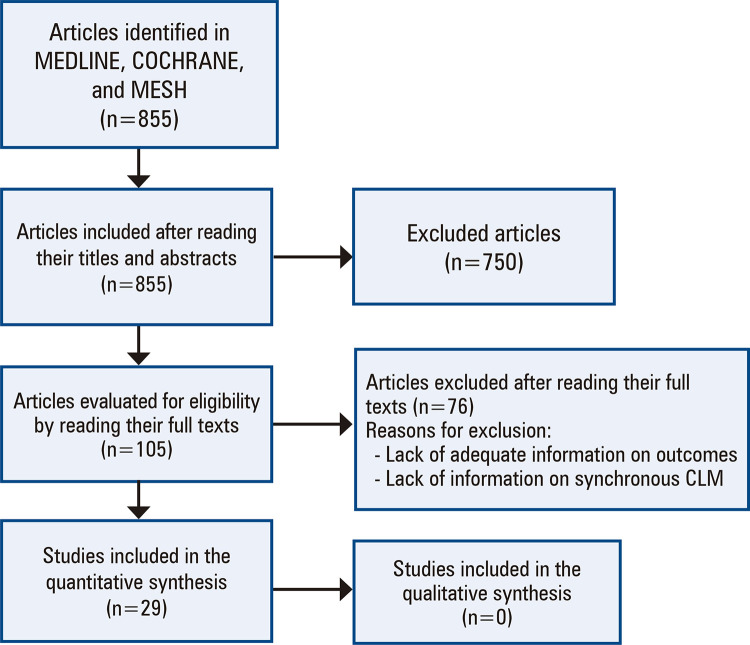
Flowchart of the methodology and search strategy of this review

This systematic review was conducted in accordance with the PRISMA recommendations.^(29)^ Approval from an ethics committee was not required for this review because it was conducted using data that are freely available in public domains (MEDLINE, EMBASE, LILACS, and Cochrane Central Register of Controlled Trials) or can be accessed without needing to contact the authors. Figure 1 shows the flowchart of the methodology and search strategy of this review.

The MEDLINE (1966 to 2022), Cochrane Central, EMBASE, and LILACS electronic databases were searched using Medical Subject Headings to identify and retrieve relevant articles. The search descriptors used were as follows: colorectal cancer or colorectal neoplasm; liver metastasis or hepatic metastasis; colorectal liver metastasis surgery; synchronous colorectal liver metastasis; hepatectomy or liver resection or hepatic resection; rectal cancer; liver-first; reverse strategy or reverse approach. Boolean operators were used to identify keyword variations and ensure they were captured during the search.

The titles of the retrieved articles were assessed and potentially eligible articles were selected. Subsequently, the abstracts of the selected articles were reviewed for further assessment of eligibility and their reference lists were searched to identify other related articles.

### Inclusion and exclusion criteria

The inclusion criteria for study selection were as follows: (a) included patients with synchronous CLM, (b) reported data on surgical events and outcomes, and (c) the longest follow-up or largest sample in cases where two or more studies were published by the same institution. The exclusion criteria were lack of information on whether the metastatic hepatocellular carcinoma was synchronous and inadequate reporting of most of the results.

### Study selection procedure

Articles that included outcomes of the liver-first approach for the treatment of synchronous CLM were included. The references of all articles considered eligible were reviewed to identify articles that may have been missed during the initial search. The identified articles were reviewed using the pre-established inclusion. The minimum prerequisite for inclusion into the present review was the evaluation of a cohort of patients with synchronous CLM who were treated using the liver-first approach.

The articles were evaluated individually by two reviewers (BMB and JW) using the predefined criteria. After the initial search, journals, case reports, editorials, duplicate studies, conference summaries, non-human studies, and studies not published in English, Portuguese, Spanish, Japanese, German, French, or Italian were excluded. Studies that described the use of systemic chemotherapy followed by liver rescue surgery for patients with initially unresectable CLM were included. Studies that focused on the proposal of a hybrid method based on a combination of liver resection ablation techniques, two-stage hepatectomy, or resection of extrahepatic metastases with the aim of meeting the criteria for resection of CLM were also included for analysis, as long as the treatment was administered with curative intent. The abstracts of the relevant studies were retrieved and subsequently reviewed for confirmation of eligibility. Thereafter, the full texts of the selected articles were reviewed methodically. Articles that described the use of the liver-first approach with intent of curing synchronous CRC and CLM were selected for review. Only articles that reported the survival outcomes (overall, short-, or long-term) of the liver-first approach were included. Studies that involved the use of liver arterial perfusion as a method of chemotherapy or radiopharmaceutical delivery were excluded. In cases where multiple reports were from the same or overlapping patient series, only the most complete or most recent study was included. The inconsistencies were mutually determined. The results of the included studies were extracted and grouped as single-arm studies without a comparison arm.

### Data extraction and critical evaluation

The two reviewers used a predefined protocol for data extraction. The data obtained included the following: title and reference information (first author, journal, and year); clinicopathological characteristics; plasma carcinoembryonic antigen (CEA) level; primary lesion site; location, number, and size of CLM; response to neoadjuvant systemic chemotherapy before resection of CLM; average number of systemic chemotherapy cycles; number of large hepatectomies; percentage of R0 resections performed; percentage of patients who completed the liver-first approach protocol; morbidity and mortality outcomes; follow-up; recurrence; disease-free survival; overall survival; and 1-, 3-, and 5-year survival outcomes.

A large hepatectomy was defined as resection of three or more Couinaud segments. Complete response was defined as total disappearance of all hepatic lesions, and partial response was defined as a 50% or more decrease in the sum of the largest diameters of the target hepatic lesions. Progressive disease was defined as a 25% or greater increase in the sum of the largest diameters of the target hepatic lesions. If the partial response or progressive disease criteria were not met, the disease was considered stable.

The two reviewers recorded the extracted data separately to minimize selection bias. Duplicate articles were removed and all discrepancies were clarified. Disagreements were resolved by the most senior reviewer.

### Outcomes of interest

The primary outcomes of interest in this review were recurrence, disease-free survival, overall survival, and one-, three-, and five-year overall survival, whereas the secondary outcomes were postoperative complications and 30-day mortality.

### Eligibility and quality assessment

Studies that included descriptions of the procedures performed in the liver-first approach and the outcomes of interest were evaluated for inclusion.

### Meta-analysis

A proportional meta-analysis was performed using the random effects restricted maximum likelihood method for the evaluation and analysis of the three- and five-year overall survival and recurrence rates. In this method, the proportion of measures is interpreted as a percentage. Cochran’s Q test, which presents the null hypothesis that the studies comprising the meta-analysis are homogeneous, was also used for analysis. Heterogeneity between studies was assessed using the I^2^ statistic, and publication bias between the studies was assessed using the Egger test.

All statistical analyses were performed using the STATA software, version 16 (Timberlake Analytics Software, NY, USA). An alpha error of 5% (0.05) was used as the statistical parameter. We sought to distinguish between the treatments by comparing them individually and grouping them by considering the articles identified in the literature.^(1-59)^

## RESULTS

A total of 855 articles were identified in the database search. After three independent reviewers screened the titles and abstracts of the identified articles, 750 were excluded. The remaining 105 articles were then thoroughly reviewed. Thereafter, 29 studies that met the selection criteria were reviewed and included in the analysis.^(29-57)^Of the 29 studies, 19 were observational studies (level of evidence: IV )^(30,33,34,36,37,41,44-52,56-59)^ and 10 were comparative retrospective cohort studies (level of evidence: III).^(29,31,32,35,38-40,42,43,53)^ No randomized controlled trial was identified in the literature search. A total of 2,499 patients with synchronous CLM who were treated using the liver-first approach were included in the 29 studies. The patient selection criteria for the liver-first approach were described in all the articles, and none of the criteria used in the studies were identical.

The mean age of the patients included in the studies was 60 years (21–86 years), and males and females accounted for 62.9% and 37.1% of the patients, respectively.^(29-31,33-38,41-57)^Twenty-one of the studies included patients with colon and rectal cancer with synchronous CLM,^(29-31,33,35,36,40-43,46-55,57)^ whereas eight studies included only patients with synchronous rectal cancer.^(32,34,37-39,44-56)^ The rectum was the primary cancer site in 1,500 patients (60.3%), whereas the colon was the primary site in 989 patients (39.7%). At diagnosis, majority (92.5%) of the patients had only liver metastases, and 7.5% had concomitant hepatic and extrahepatic CRC metastases.^(30,34-40,44-48,51-54,56,57)^ The mean plasma CEA level reported in 15 studies was 100.8ng/ml (1–8,456 ng/ml),^(29-31,33,34,37-41,44,47,52,53,57)^ whereas the mean diameter of the resected liver metastases was 3.7cm (1–20 cm).^(29-31,33-37,39-42,44,45,47,49,52,53,55-57)^

Regarding patients who were initially treated using the liver-first approach, only two studies^(48,56)^did not present data regarding the end of the liver-first approach protocol. Of 1,677 patients, 1,352 (80.6%) completed the protocol, whereas 325 patients (19.4%) did not, primarily due to advanced disease.^(29-45,47-53,55-57)^

The number and duration of preoperative chemotherapy regimens varied among the reviewed studies. The mean number of neoadjuvant chemotherapy cycles for hepatic metastases was six (3–12 cycles).^24,31-34,36^ The chemotherapy agents used were 5-fluorouracil, leucovorin, oxaliplatin, irinotecan, bevacizumab, and cetuximab, either alone or in combination. Chemotherapy was administered before and after liver resection.

The effects of chemotherapy on the hepatic parenchyma were reported in only two studies.^(53,57)^ The presence of steatohepatitis and fibrosis was evaluated and graded according to the METAVIR score. In the two studies, 41 (38.6%) patients out of 106 had fibrosis after chemotherapy.

Radiological response to neoadjuvant treatment, evaluated according to the Response Evaluation Criteria In Solid Tumors, was reported in 12 studies.^(34,39-41,44,46-48,50,53,56,57)^A total of 852 patients were assessed in these studies, and 21 (2.5%) of them showed complete response to preoperative chemotherapy, 666 (78.2%) showed partial response, 140 (16.4%) had stable disease, and 25 (2.9%) had progressive disease.

Table 2 shows the interventions administered for patients with synchronous hepatic metastases of colorectal cancer in the 29 studies analyzed in this review. Of 2,298 (92%) patients who underwent hepatic resection, 811 (35.3%) underwent major hepatectomies.^(31,33,35-38,40-43,46-51,53-57)^ R0 resection was performed for 936 (69.2%) of the 1,352 patients who completed the protocol.^(29-45,47-53,55-59)^Other surgical procedures performed or analyzed in the studies included portal vein embolization, cryotherapy, radiofrequency ablation, two-stage hepatectomy, and excision of extrahepatic metastases.

**Table 2:** Interventions administered to patients with synchronous hepatic metastases of colorectal cancer in the 29 studies included in this review

	End of protocol n (%)	Average number of chemotherapy cycles	Hepatic resections, n (%)	R0 resection, n (%)
Esposito et al.^(29)^ (n=66)	63 (95.4)	NA	66 (100)	56 (89.9)
Mentha et al.^(30)^ (n=35)	30 (85.7)	4 Cycles	30 (100)	30 (85.7)
Brouquet et al.^(31)^ (n=27)	27 (100)	7 Cycles	27 (100)	23 (85)
van der Pool et al.^(32)^ (n=20)	20 (100)	6 Cycles	20 (100)	NA
de Jong et al.^(33)^ (n=22)	18 (81.8)	6 Cycles	21 (95.4)	20 (95.2)
Ayez et al.^(34)^ (n=42)	31 (73.8)	5 Cycles	40 (95.2)	31 (74)
Mayo et al.^(35)^ (n=28)	28 (100%)	NA	28 (100)	8 (28.6)
de Rosa et al.^(36)^ (n=37)	25 (67.5)	6 Cycles	30 (81)	17 (56.7)
Buchs et al.^(37)^ (n=34)	33 (97)	3 Cycles	33 (97)	32 (93.9)
Sabbagh et al.^(38)^ (n=10)	5 (50)	NA	8 (80)	5 (50)
Tanaka et al.^(39)^ (n=10)	2 (20)	6 Cycles	10 (100)	5 (50)
Okuno et al.^(40)^ (n=12)	12 (100)	12 Cycles	12 (100)	6 (50)
Wang et al.^(41)^ (n=18)	16 (88.9)	3 Cycles	18 (100)	18 (100)
Welsh et al.^(42)^ (n=98)	82 (83.6)	NA	98 (100)	91 (93)
Valdimarsson et al.^(43)^ (n=246)	162 (65.8)	NA	246 (100)	173 (70.3)
Nierop et al.^(44)^ (n=129)	90 (70)	4 Cycles	117 (90.6)	90 (70)
de Jong et al.^(45)^ (n=92)	70 (76.1)	NA	86 (93.4)	NA
Giuliante et al.^(46)^ (n=552)	NA	NA	541 (98)	NA
Fonollosa et al.^(47)^ (n=88)	75 (85.2)	8.5 Cycles	75 patients (85.2)	46 (61.3)
Carbone et al.^(48)^ (n=26)	15 (42.3)	NA	26 (100)	18 (72)
Frühling et al.^(49)^ (n=163)	163 (100)	NA	163 (100)	132 (81)
Raoux et al.^(50)^ (n=26)	26 (100)	NA	26 (100)	19 (73)
Reding et al.^(51)^ (n=7)	7 (100)	NA	7 (100)	4 (57)
Harufumi et al.^(52)^ (n=141)	91 (64.5)	NA	141 (100)	NA
Giammauro et al.^(53)^ (n=62)	49 (79)	NA	62 (100)	46 (74.2)
Vallance et al.^(54)^ (n=270)	NA	NA	137 (50.7)	NA
Ramia et al.^(55)^ (n=149)	131 (88)	6 Cycles	149 (100)	NA
Labori et al.^(56)^ (n=45)	40 (89)	4 Cycles	45 (100)	40 (89)
Pasquier et al.^(57)^ (n=44)	41 (93)	6 Cycles	44 (100)	26 (61)

SC: systemic chemotherapy; NA: not available.

The average postoperative morbidity rate reported in the studies was 30.6% (0-80%). Minor or major complications evaluated according to the Clavien-Dindo classification occurred in 566 (24.6%) of the 2,298 patients who underwent hepatic resection.^(31,33,35-38,40-43,46-50,53-59)^ The mean perioperative mortality rate was 5.7% (0-76.6%).^(29-31,33-38,40-42,44-59)^ For a total of 2,252 patients, the mean follow-up duration was 40.5 months (12–120 months),^(30-32,34-38,40-54,56-59)^ the disease-free survival was 16.6 months (3–51 months),^(29,31,33-36,38,40-42,45-48,50,51,56,57)^ and the recurrence rate was 51.5% (14.7–100%).^(29-33,35-37,39-42,45-47,50,51,55-57)^ In addition, the mean overall survival was 46.4 months (12–64 months),^(30-36,38,42,44-46,49,50,52,53,56,57)^ the one-year overall survival was 85.9% (11.1–100%),^(29,30-37,40,41,43,47-54,56,57)^ the three-year overall survival was 62.9% (30.4–88%),^29-31,33-37,40,41,45-53,56,57^and the five-year overall survival was 47.4% (14.3–87.5%)^(29-32,34,35,37,42,43,45-54,56,57)^ (Table 3).

**Table 3:** Primary and secondary outcomes (one-, three-, and five-year overall survival and morbidity, mortality, and recurrence) in patients with synchronous hepatic metastases of colorectal carcinoma treated using the liver-first approach

	1-year OS (%)	3-year OS (%)	5-year OS (%)	Morbidity, n (%)	Mortality, n (%)	Recurrence, n (%)
Esposito et al.^(29)^ (n=66)	100	88	72	30 (45.5)	-	36 (54.5)
Mentha et al.^(30)^ (n=35)	100	60	31	5 (17)	3 (1)	20 (57.1)
Brouquet et al.^(31)^ (n=27)	NA	79	39	8 (31)	1 (4)	19 (70)
van der Pool et al.^(32)^ (n=20)	NA	NA	67	6 (30)	N.A.	4 (20)
de Jong et al.^(33)^ (n=22)	74.2	41.1	NA	6 (27.3)	-	6 (33.3)
Ayez et al.^(34)^ (n=42)	100	79	67	10 (23)	-	NA
Mayo et al.^(35)^ (n=28)	89	60	44	11 (39.3)	-	12 (42.9)
de Rosa et al.^(36)^ (n=37)	65.9	30.4	NA	12 (40)	1 (4.2)	13 (52)
Buchs et al.^(37)^ (n=34)	81.6	68	52.5	9 (27.3)	-	5 (14.7)
Sabbagh et al.^(38)^ (n=10)	NA	NA	NA	2 (20)	1 (10)	NA
Tanaka et al.^(39)^ (n=10)	11.1	NA	NA	4 (40)	NA	9 (90)
Okuno et al.^(40)^ (n=12)	100	87.5	87.5	5 (41.6)	-	7 (58.3)
Wang et al.^(41)^ (n=18)	94.4	44.8	NA	4 (22.2)	-	16 (88.9)
Welsh et al.^(42)^ (n=98)	NA	NA	44	10 (10)	2 (2)	30 (37)
Valdimarsson et al.^(43)^ (n=246)	100	NA	49%	NA	NA	NA
Nierop et al.^(44)^ (n=129)	NA	NA	NA	8 (6)	1 (0.7)	NA
de Jong et al.^(45)^ (n=92)	NA	48.5	33.1	29 (31.5)	3 (3.3)	36 (51.4)
Felice et al.^(46)^ (n=552)	NA	65.9	51.4	171 (31.1)	26 (4.8)	203 (36.8)
Fonollosa et al.^(47)^ (n=88)	95	74	53	17 (22.6)	-	57 (76)
Carbone et al.^(48)^ (n=26)	74	54	36	10 (38.4)	-	NA
Frühling et al.^(49)^ (n=163)	90.8	61.9	43.6	132 (80.9)	1 (0.6)	NA
Raoux et al.^(50)^ (n=26)	96	74	50	13 (50)	1 (3.8)	17 (65)
Reding et al. ^(51)^ (n=7)	71.4	58	14.3	NA	1 (14)	7 (100)
Harufumi et al.^(52)^ (n=141)	75.2	41.8	23.4	19 (13.5)	108 (76.6)	NA
Giammauro et al.^(53)^ (n=62)	95	76	55	-	14 (22.6)	47 (75.8)
Vallance et al.^(54)^ (n=270)	100	NA	58	NA	-	NA
Ramia et al.^(55)^ (n=149)	NA	NA	NA	17 (11.4)	1 (0.7)	NA
Labori et al.^(56)^ (n=45)	97.7	71.1	33.3	5 (11.1)	-	30 (75)
Pasquier et al.^(57)^ (n=44)	93	59	39	23 (52)	-	11 (25)

OS: overall survival; NA: not available.

As shown in table 3, data on one-, three-, and five-year overall survival and disease-free survival were not reported in some studies, and in others, the information presented differed. Therefore, to avoid information bias or data estimation bias, we chose not to include this information in our analysis.

### Results of the meta-analysis

**Figure 2:**
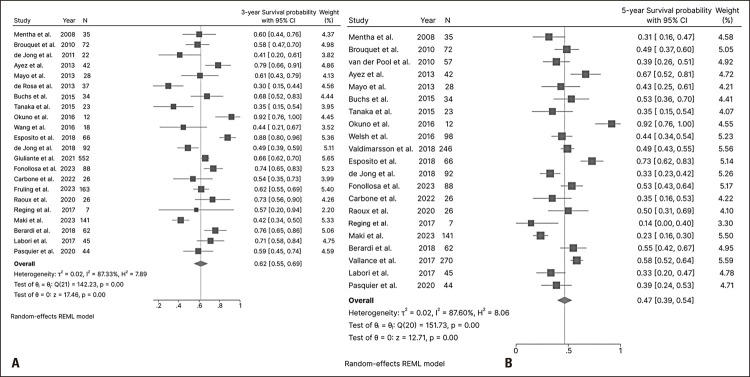
Three- and five-year overall survival rates of patients who completed treatment administered using the liver-first approach

The three- and five-year overall survival rates reported in the studies were 62% (55–69%, 22 studies) (Figure 2A) and 47% (39–54%, 21 studies) (Figure 2B), respectively. Significant heterogeneity was observed among studies in both analyses (I^2^=87.3%, p<0.01 and I^2^=87.6%, p<0.01, respectively).

**Figure 3:**
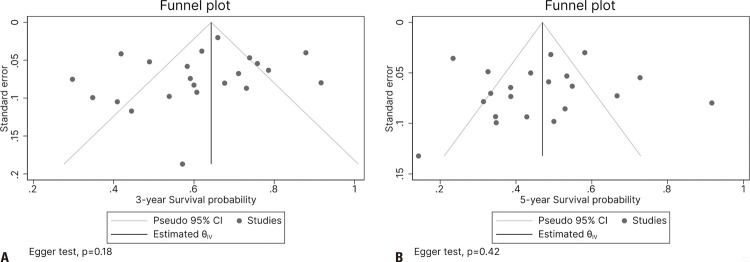
Funnel plots of publication bias for three- and five-year overall survival rates among the studies included in the meta-analysis

Publication bias was assessed using funnel plots, and the results showed no significant publication bias in the studies. In addition, there was no publication bias regarding three- and five-year overall survival rates among the studies (p=0.18 and p=0.42, respectively) (Figures 3A and 3B, respectively).

**Figure 4:**
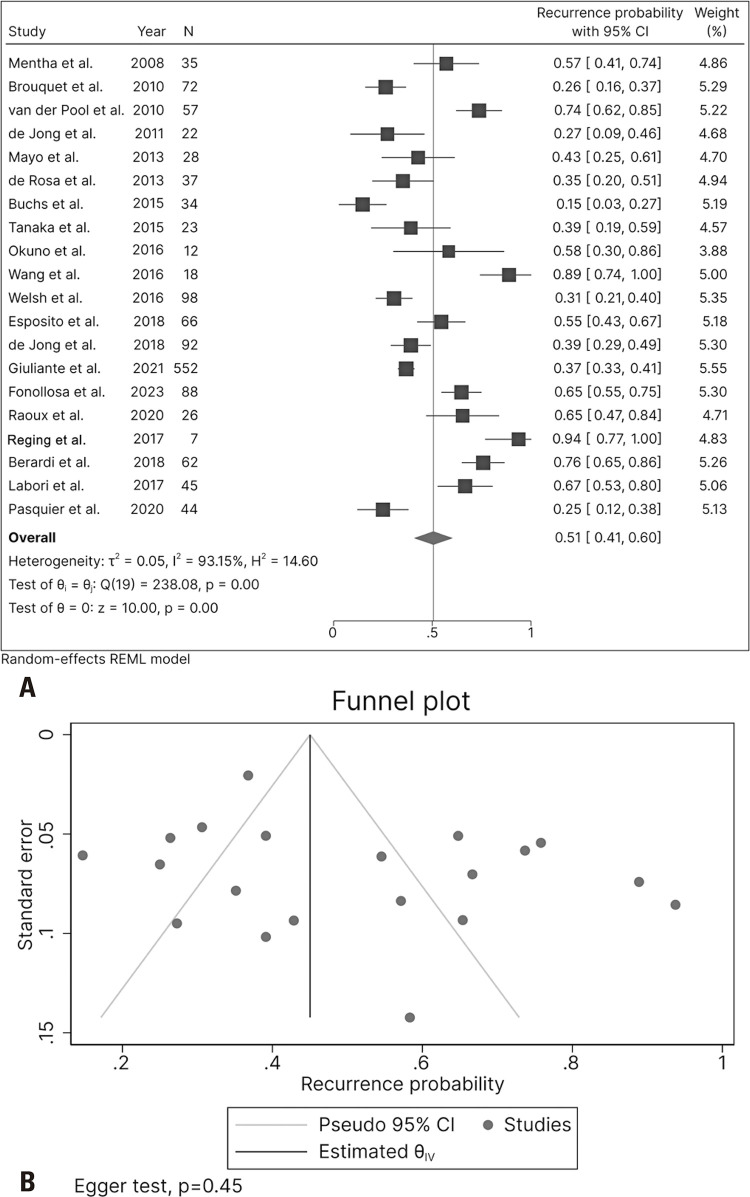
Forest and funnel plots of recurrence rate in patients who completed the liver-first approach protocol

The recurrence rate reported in 20 studies was 51% (41–60), and the included studies showed significant heterogeneity (I^2^=93.15, p<0.01) (Figure 4A) but no publication bias (Egger test, p=0.45) (Figure 4B).

Additional analysis was performed according to the types of treatment approach (primary [classical], liver-first [reverse], and combined) analyzed in the included studies to estimate the relationship between three- and five-year survival rates and treatment approaches, as well as that between recurrence rate and treatment approaches. There were no statistically significant differences in three-year survival (p=0.31), five-year survival (p=0.31), and recurrence (p=0.09) rates between groups.

The three- and five-year survival rate and recurrence rate were 70% (45 – 96, two studies), 44% (29 – 58, seven studies) and 65% (53 – 77, five studies), respectively, for the classical approach; 65% (51 – 79, two studies), 61% (44 – 77, two studies), and 52 (47 – 57, two studies), respectively, for the combined approach, and 80 (67 – 92, two studies), 51% (33 – 69, five studies), and 65% (47 – 83, five studies), respectively, for the reverse approach.

## DISCUSSION

Liver metastasis of colorectal cancer is a major clinical issue. The liver is the main site of metastasis in CRC, and although two-thirds of patients show extrahepatic spread, others have isolated liver disease. The treatments available for liver metastases of CRC include surgical resection, thermal ablation, regional hepatic intra-arterial chemotherapy, chemoembolization, radioembolization, and radiation therapy, including stereotactic radiation therapy. Among these treatments, surgical resection remains the gold standard because it is associated with better long-term disease-free survival.

The optimal timing and sequence of surgical resection of CRC remain controversial.^(43)^ However, decision-making regarding the timing of the resection depends on the patient’s symptoms and disease burden. Patients who have complications of primary CRC, such as bleeding, obstruction, or perforation, should initially undergo colorectal tumor resection. The liver-first approach can delay the resection of CRC and increase the risk of developing complications. Some studies have shown that the rates of bleeding, obstruction, and perforation associated with the liver-first approach are as high as 20% each.^(27,45)^ Asymptomatic patients with primary CRC may undergo simultaneous or staged resection depending on the extent of liver involvement. In addition, patients with CRC in a favorable location (*e.g*., the right colon) and limited hepatic metastases may require simultaneous resection of CRC and liver metastases. Moreover, patients who are treated with neoadjuvant chemotherapy may benefit from a two-stage hepatic approach, whereas with locally advanced rectal cancer (T4 and/or bulky tumor or extensive lymph node disease) may benefit from intense preoperative induction chemotherapy followed by chemoradiotherapy instead of isolated chemoradiotherapy.^(27,45,58)^If such patients have synchronous and potentially resectable liver metastases, they may be treated using the liver-first approach after four months of induction chemotherapy. Thereafter, a new chemotherapy therapy is administered for another two months, and 4–8 weeks after the end of the chemotherapy, colorectal resection can be performed.^(58)^

In patients with synchronous CRC and CLM, preoperative chemotherapy administered using oxaliplatin-and/or irinotecan-based regimens induces an important histological response in the CRC.^(3,4,8,10)^ This response is significantly associated with CLM response and leads to conversion of non-resectable disease to resectable disease (downstaging).^(12,14,16,23)^

Resection of CLM is contraindicated for patients with more than four metastases, extrahepatic disease, or less than 1 cm of free margin of resection.^(3,5,11,13)^ However, some studies have shown that patients with these clinicopathological factors can achieve long-term survival after liver resection and should not be excluded as candidates for surgery.^(4,5,19)^

A collective analysis was conducted in the present review. In this analysis, the data of all the included studies were grouped together as if obtained from a single cohort. It should be noted that the studies included in this review showed a great deal of heterogeneity in their designs; therefore, their different results must be taken into account. Moreover, the cohorts in the few previous studies on the use of the liver-first approach may not be representative of the population of patients with synchronous CLM. Notably, no randomized controlled trial on the different treatment options for CLM has been conducted to date. In addition, a published series^(29-57)^ showed differences in patient age, CRC site, and liver metastasis characteristics (size, number, and distribution) among studies. Furthermore, there are significant differences in the existing chemotherapy regimens, particularly with respect to the new biological agents. These variables induce heterogeneity and variability, making drawing of any potential conclusion challenging.

The lack of uniformity in the definition of unresectability is an important limitation in the critical evaluation of the outcomes of resection of CLM that was initially untreatable after neoadjuvant chemotherapy. Standardization of unresectability criteria will certainly facilitate better understanding of the role of neoadjuvant chemotherapy in the treatment of CLM and ensure that the best treatment is administered to potential resection candidates. Moreover, widespread adoption of uniform definitions will facilitate the interpretation of the results of future studies.

Only two of the studies analyzed in this review included objective evaluation of liver damage caused by chemotherapy, such as fibrosis, steatohepatitis, and sinusoidal damage. These unfavorable outcomes may increase the risk of liver failure, particularly after extended hepatic resection.

Successful completion of treatment administered using the liver-first approach depends on the achievement of downstaging in response to neoadjuvant chemotherapy.^(32-34,37)^In the studies included in this systematic review, the liver-first was utilized in 80.6% of cases, with the results indicating a high response rate. The reasons for therapy failure included disease progression, new extrahepatic disease, postoperative death after hepatic resection (30 days), and loss to follow-up.^(36,38,41)^

Most of the patients in the included studies underwent extensive liver resection. Specifically, 936 (69.2%) of the 1,352 patients who completed the protocol underwent R0 resection.^(29-45,47-53,55-59)^ The post-hepatectomy morbidity rate was 30.6%, whereas the postoperative mortality rate (30 days) was 5.7%. These outcomes indicated that chemotherapy for downstaging did not prevent patients from requiring extensive liver resection with a high chance of achieving R0 resection. Notably, the mean three- and five-year overall survival rates were 62.9% and 47.4%, respectively.

The present study revealed that use the liver-first approach for the treatment of patients with CRC and synchronous CLM is associated with low perioperative morbidity and mortality, as well as satisfactory survival outcomes.^(29-45,48-59)^ These results are comparable with those of studies of patients who were treated using the classical approach (resection of CRC and subsequent resection of metastatic hepatocellular carcinoma).^(29,31- 33,35,38-40,42,43,46,48-52,54)^ In this review, we compared the survival outcomes reported in retrospective and comparative cohort studies that involved analyses of the classical approach and the liver-first approach. In these studies, the mean disease-free survival of patients treated using the classical approach was 28.5 months and their one-, three-, and five-year overall survival rates were 99.5%, 76.5%, and 50.9%, respectively.^(29,31-33,35,38-40,42,43)^ To avoid information and data estimation biases, we chose not to consider the secondary results of studies with data discrepancies.^(31,32,35,38-40,42,43,46)^

The results observed in this review support liver resection as the gold standard surgical treatment option for CRC, as well as the curative treatment of choice for CLM^(34,37,39,40)^ In addition, the meta-analysis, including the survival curves for the patients evaluated in the reviewed studies, confirmed the abovementioned findings. Zeyara et al.^(59)^ conducted a meta-analysis of 17 studies on the overall survival (OS) and clinicopathological data of patients (n=1041) treated using the liver-first approach. The authors observed an average liver-first approach completion rate of 80% and a median overall survival of 45 and 13 months for the liver-first approach completion and non-completion groups, respectively. Furthermore, the results of the meta-analysis indicated that the liver-first approach completion group had a significant survival benefit. The main cause of non-completion (76%) was the progression of liver disease before resection of the primary colorectal tumor.

This review has some limitations. First, all the included studies were retrospective and based on odds ratio measures, making it difficult to perform a hazard ratio analysis, which despite being less susceptible to bias, behaves similarly to odds ratios. Second, most of the studies analyzed in this review included a small number of patients with metastatic tumors that were initially resectable. Third, there was substantial heterogeneity in the treatment strategies employed in the studies. This heterogeneity is attributable to differences in interventions, such as type of surgery and use of ablative techniques, outcome evaluations, and number of patients. Furthermore, there was significant heterogeneity in the five-year overall survival outcomes of the patients treated using the liver-first approach.

## CONCLUSION

This results of this review indicate that chemotherapy is important for disease control and downstaging in patients with a high liver tumor load. Initial liver resection should be considered for these patients, as this can influence their long-term survival. The liver-first approach offers the possibility of complete resection of colorectal liver metastases and guaranteed overall survival benefit if completed. Notably, non-completion of the liver-first approach is associated with a higher number of liver metastases. These findings indicate that the decision regarding the timing of colorectal cancer and colorectal liver metastases resections should be personalized for each patient.

This review is unique and makes a valuable contribution to the literature because it addresses a controversial subject that has not been evaluated in randomized controlled trials, highlighting the growing need for randomized controlled trials and multicenter studies as the next step in researching the liver-first approach for the treatment of colorectal liver metastases.
